# An inverse relationship between fitness and secretion efficiency in a gram-positive bacterium

**DOI:** 10.1093/pnasnexus/pgaf131

**Published:** 2025-04-28

**Authors:** Kamilla Wiull, Morten Kjos, Vincent G H Eijsink, Geir Mathiesen

**Affiliations:** Faculty of Chemistry, Biotechnology and Food Science, NMBU—Norwegian University of Life Sciences, Ås 1433, Norway; Faculty of Chemistry, Biotechnology and Food Science, NMBU—Norwegian University of Life Sciences, Ås 1433, Norway; Faculty of Chemistry, Biotechnology and Food Science, NMBU—Norwegian University of Life Sciences, Ås 1433, Norway; Faculty of Chemistry, Biotechnology and Food Science, NMBU—Norwegian University of Life Sciences, Ås 1433, Norway

**Keywords:** heterologous protein secretion, LPxTG protein, HaloTag, signal peptide, bacterial fitness

## Abstract

*Lactiplantibacillus plantarum* and other lactic acid bacteria are emerging as promising candidates for mucosal delivery of surface-displayed antigens. However, producing secreted heterologous proteins and anchoring these using LPxTG anchors can significantly reduce bacterial fitness. To understand the underlying mechanisms and limiting factors, we analyzed 11 recombinant *L. plantarum* strains expressing the HaloTag reporter protein with the same LPxTG anchor but varying signal peptides. By labeling the reporter protein with fluorescent ligands, this approach allowed simultaneous detection of correctly folded intracellular and extracellular protein, revealing signal peptide-dependent variation in the relative amounts of intra- and extracellularly folded HaloTag. Furthermore, this analysis uncovered an unexpected correlation between secretion efficiency and bacterial fitness. Strains with better growth showed more premature intracellular folding and reduced protein translocation and surface display. Conversely, strains with a higher fraction of surface-displayed protein, i.e. strains with greater secretion efficiency, exhibited impaired growth, likely due to increased interactions between the signal peptide and the secretion machinery, leading to secretion overload. Correlation analyses and confirmation of observed correlations by mutational studies of the signal peptides showed that signal peptide hydrophobicity is positively correlated with higher secretion efficiency but is accompanied by a trade-off in fitness. These findings underscore the critical role of signal peptides in balancing protein secretion and bacterial viability, offering valuable insights for optimizing protein secretion and anchoring in gram-positive bacteria

**Significance Statement**

This study provides insights into the role of signal peptides in the secretion and surface display of heterologous proteins in the model bacterium *Lactiplantibacillus plantarum*, a promising gram-positive candidate for mucosal vaccine delivery. The study uncovers an unexpected trade-off between effective protein secretion and bacterial fitness. Furthermore, the identification of a positive correlation between signal peptide hydrophobicity and secretion efficiency, achieved through computational and mutational approaches, provides important information for the rational design and selection of signal peptides. This work advances the understanding of signal peptide functionality in gram-positive bacteria and provides key insights that may contribute to a more refined selection of signal peptides for improved production and secretion of heterologous proteins.

## Introduction

The exploitation of bacteria to produce enzymes and antigens has been extensively investigated owing to their genetic simplicity and rapid growth (1). The most used species for this purpose are *Bacillus subtilis* and *Escherichia coli* (2), however, *Lactiplantibacillus plantarum* has also been extensively used for the expression of heterologous proteins (3–5) and is an especially attractive candidate for the developing mucosal vaccines (6, 7). An important feature for development of bacteria as vaccine delivery vehicles is to efficiently display a heterologous protein on the bacterial surface without reducing bacterial fitness. A commonly used protein motif for anchoring antigens to the peptidoglycan cell wall in gram-positive bacteria is the C-terminal LPxTG motif (8, 9). After translocation, LPxTG proteins are processed by a membrane-bound sortase A, which cleaves the LPxTG motif at the threonine (T) residue, and covalently attaches it to the peptidoglycan layer (10). The length of the anchoring sequence, i.e. the length of the linker region connecting the C-terminus of the to-be-displayed protein to the LPxTG sequence, varies.

Most of the secreted proteins of *L. plantarum* are exported out of the cell by the Sec secretion machinery, including proteins carrying the LPxTG motif (11, 12). Several proteins constitute the Sec secretion machinery. The membrane-bound translocon channel consists of a complex of SecY, SecE, and SecG (SecYEG), while an ATPase called SecA, located on the cytoplasmic side of the translocon, drives the translocation (13). Only unfolded proteins can be secreted through the SecYEG translocon, substantiating the need for effective secretion systems that, next to targeting the protein through the translocon, prevent premature folding. Chaperones play a crucial role in preventing premature folding, and it is noteworthy that *L. plantarum* encodes most of the chaperones known for their importance in *E. coli*. These include trigger factor (tf), DnaK, DnaJ, and GrpE (also known as the DnaJKE chaperone) and GroEL/ES (14).

The N-terminal signal peptides that direct proteins to the Sec secretion machinery are cleaved off during translocation across the cell membrane. The cleavage is mediated by either signal peptidase I (SPI) or signal peptidase II (SPII), depending on signal peptide characteristics. The most extensively studied extracellular proteins in bacteria are those containing a SPI signal peptide, which includes secreted proteins and proteins harboring an LPxTG-peptidoglycan anchor domain (15–18). The SPI signal peptides are typically 15–30 amino acids long and exhibit limited sequence conservation. However, they exhibit three distinct regions; the N, H, and C regions (19). At the N terminus, the N region (5–8 residues) typically includes several positively charged residues. The H region is composed of hydrophobic residues (8–12 residues), whereas the polar C region typically comprises 5–7 residues followed by the A-X-A cleavage site motif that is recognized by SPI (2, 20).

The two main pathways for Sec secretion in prokaryotes are termed the co- and posttranslational pathways. Most secreted proteins follow the posttranslational SecA pathway (21). In the SecA pathway, translation and translocation are separate processes and signal peptides bind to the preprotein binding domain of SecA (22, 23). Conversely, in the co-translational pathway, the signal recognition particle (SRP) ribonucleoprotein is recruited during translation. The SRP has a high affinity for hydrophobic signal peptides and is involved in the secretion of a subset of secreted proteins as well as in the translocation of membrane proteins. Membrane proteins carry a so-called signal anchor sequence, i.e. a hydrophobic signal peptide that is not cleaved off during translocation (24, 25). It has been shown that binding of the nascent protein to SRP prevents protein aggregation and premature folding (26). Studies with *E. coli* have shown that the co-translational pathway is important for translocating overexpressed recombinant cytoplasmic proteins into the periplasm, which can be achieved by using a highly hydrophobic signal peptide (27).

Optimization of secretion has been investigated mostly using secreted proteins without an anchor motif. Brockmeier et al. (16) systematically screened all predicted SPI signal peptides from *B. subtilis* by fusing them to a cutinase or an esterase. Pulse-chase experiments showed that secretion yields correlated to varying efficiencies of SP processing. A study of nuclease secretion in *Lactococcus lactis* showed that homologous signal peptides resulted in more efficient processing of the signal peptide and higher secretion (28). An investigation of the performance of all native predicted SPI signal peptides in the secretion of a nuclease in *L. plantarum* WCFS1, revealed huge variation in the efficacy of signal peptides (15).

The studies discussed above show that the signal peptide has a huge impact on secretion efficiency and that this impact is partly dependent on the nature of the protein to be secreted. Importantly, these studies do not address the signal peptide-dependent impact of heterologous protein expression on bacterial growth fitness, nor do they unravel particular features of the signal peptides that determine secretion and fitness. It is well known that overexpression of secreted heterologous proteins in gram-positive bacteria may lead to secretion stress and reduce bacterial fitness (29–32), but the underlying causes are not understood. As an example, studies aimed at producing LPxTG-anchored antigens in *L. plantarum* have yielded strains that, while successful in terms of actual surface display of the antigen and even in vivo immunological responses, show clear growth defects (29, 33, 34). To gain more insight into these matters, we have generated 11 *L. plantarum* strains expressing an LPxTG-anchored HaloTag reporter protein using the exact same LPxTG anchor but 11 different signal peptides. By independently and simultaneously determining intracellular and extracellular levels of the folded HaloTag protein, we investigated how signal peptide-dependent variation influences growth performance and the levels of surface-anchored proteins. Furthermore, a correlation between the structural features of the signal peptide and its performance was demonstrated by analyzing the effects of site-directed mutations that alter signal peptide hydrophobicity.

## Results

### The signal peptide affects growth performance during heterologous expression of LPxTG-anchored proteins

Previous studies had shown that fusion of the LPxTG anchor cwa2578 to the *Mycobacterium tuberculosis* hybrid antigen Ag85B-ESAT-6 (AgE6) leads to surface display of the antigen and immunological responses in mice, but that the producer strain grows poorly (29, 34). In order to determine whether the growth inhibition of the producer strain is related to the nature of the LPxTG anchor, we fused various predicted LPxTG anchors derived from *L. plantarum* with an N-terminal homologous signal peptide (sp3050). Specifically, we replaced the LPxTG anchor (cwa2578) in the previously described plasmid pLp_3050_Ag85B:ESAT6cwa2 (34) with six LPxTG anchors derived from *L. plantarum* (Table S1). The length of the C-terminal anchoring sequences upstream of the glycine of the LPxTG motif varied from 99 to 263 residues (Table S1). Expression of the gene fusion was under control of an SppIP-inducible promoter, and maximal gene expression was achieved by addition of 25 ng/mL of the inducing peptide SppIP to the growth medium in all samples (35). Replacing the cwa2578 anchor did not markedly improve bacterial growth (Fig. S1A and B). All strains still grew much worse than the control strain (“Cyt”) and several of the strains with new anchors essentially stopped growing after induction. Nonetheless, flow cytometry analysis confirmed surface display of the antigen in all six alternative expression strains (Fig. S1C). Because the strain with the cwa3001 anchor exhibited slightly better growth compared with the strain with the cwa2578 anchor, we selected the cwa3001 anchor for further work, turning our attention to the role of the signal peptide.

**Fig. 1.**
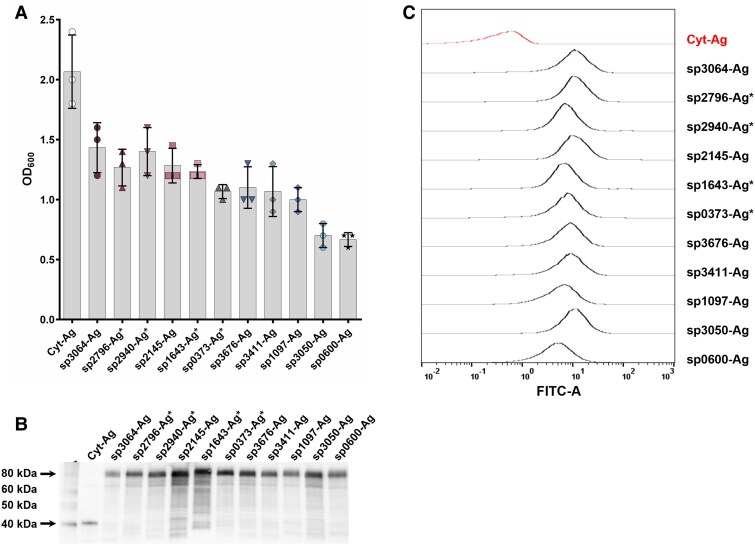
Characterization of recombinant *L. plantarum* strains expressing AgE6. In the strain labeled “Cyt-Ag,” the antigen is directed to the cytoplasm; in the other strains, the antigen is anchored to the surface through the cwa3001 anchor and secreted using one of 11 different signal peptides (sp). A) The OD_600_ measured 3 h after induction with 25 ng/mL SppIP. The error bars show the SD of three biological replicates; all data points are shown. B) The presence of AgE6 in ∼280 ng of cell-free protein extract from the various strains analyzed by western blotting. The two arrows indicate the AgE6 proteins; the anchored protein has a larger apparent size than the cytoplasmic protein due to the presence of the anchor. C) Flow cytometry analysis of surface-displayed AgE6 on the recombinant strains using an ESAT-6 specific primary antibody and a FITC-conjugated secondary antibody. The *x*-axis shows the detected FITC signal. A total of 30,000 events were analyzed for each strain. The strains marked with an asterisk in A–C have a signal peptide derived from an LPxTG protein.

Using a construct with the cwa3001 anchor, we exchanged the N-terminal signal peptide while keeping the anchor sequence constant. We selected an additional 10 predicted signal peptides derived from *L. plantarum* (Table S1), of which four originated from genes encoding LPxTG proteins (sp2940, sp2796, sp0373, and sp1643). The remaining seven signal peptides (sp3050 + six new ones) originated from proteins predicted to be secreted without any predicted anchor sequence. Of note, these 11 signal peptides were selected based on a previous study in which their functionality had been assessed experimentally, with the aim of selecting signal peptides that showed varying secretion performance. We assessed the growth, production, and surface exposure of the AgE6 antigen in the 11 recombinant strains. Despite the signal peptides being the only difference between the strains, they displayed markedly varying growth performance, with several of the new signal peptides yielding considerably improved growth compared with sp3050-Ag (Fig. 1A). The western blot (Fig. 1B) and flow cytometry analyses (Fig. 1C) confirmed that all strains produced surface-localized antigen, demonstrating the functionality of all selected signal peptides.

### Growth is inversely correlated with the level of surface-displayed protein

To allow more comprehensive analyses of protein secretion and the growth effects associated with overproduction of a secreted anchored protein, we exchanged the AgE6 antigen with the HaloTag reporter protein (36) in all 11 strains, as well as in the “Cyt” control strain in which the protein is directed to the cytoplasm. Folded HaloTag can be detected by the addition of different fluorescent ligands with varying spectral properties that bind covalently to the protein. By staining the HaloTag with both a cell-impermeable ligand (AlexaFluor 488) and a permeable (TMRDirect) ligand, extra- and intracellular folded protein can be detected in intact bacteria simultaneously. While being used for single-molecule detection in gram-negative *Salmonella enterica* (37), the HaloTag has, to our knowledge, not previously been used in gram-postive bacteria, let alone for dual detection of intracellular and surface-displayed proteins. Because the LPxTG anchor retains the secreted protein on the bacterial surface, the analysis of secretion efficiency is simplified compared with proteins that are secreted to the supernatant.

**Fig. 2.**
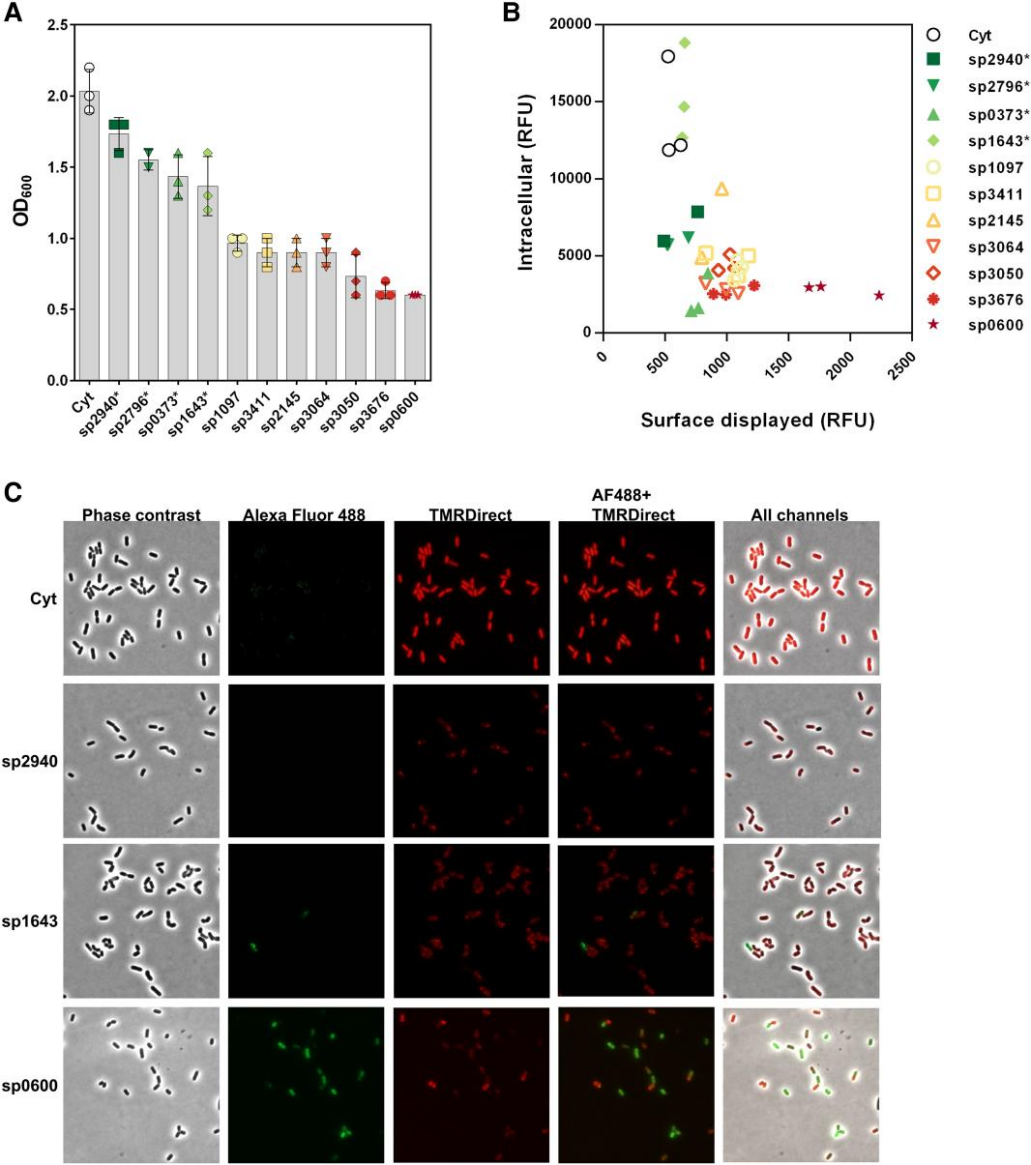
Growth (A) and an *xy* plot showing single-cell analysis (B) of recombinant *L. plantarum* strains expressing HaloTag anchored to the bacterial surface using the cwa3001 anchor and one of 11 different signal peptides. A) The OD_600_ was measured 3 h after the induction of gene expression with 25 ng/mL SppIP. The error bars represent the SD of three biological replicates and all data points are shown. B) The *y*-axis shows relative fluorescent unit (RFU) of TMRDirect-tagged intracellular HaloTag, and the *x*-axis shows RFU of AlexaFluor 488-tagged surface-displayed HaloTag. At least 500 cells were analyzed per strain. The color of the strain indicator in A and B (three data points for each strain, except sp2940 and sp2145, which have only two data points, in B) corresponds to the growth performance of the strain (green, best → red, worst). C) Representative micrographs for four of the recombinant HaloTag strains (Cyt, sp2940, sp1643, and sp0600). Micrographs of the phase contrast, the channel detecting AlexaFluor 488 on the bacterial surface and the channel detecting intracellular TMRDirect, as well as an image with all three channels merged are shown.

Similar to the AgE6-producing strains, the strains encoding HaloTag, all with the same LPxTG anchor but with different signal peptides, showed considerable variation in growth (Fig. 2A). All 11 strains did grow after induction, as shown by the full growth curves provided in Fig. S2. A clustered dendrogram analysis shows that the 11 strains can be divided into three groups based on their growth performance (Fig. S3): sp2940–sp1643 (four strains) forms the group of the “best growers” (green indicators in Fig. 2), sp1097–sp3064 (four strains) shows intermediate growth (yellow-light orange indicators), while sp3050–sp0600 (three strains) gives the lowest growth performance (red indicators).

The secretion efficiency in each strain was investigated by labeling live bacteria with cell-impermeable AlexaFluor 488 and permeable TMRDirect. Single-cell analysis of fluorescence microscopy images using MicrobeJ (see Materials and methods) showed that the four fastest-growing strains have low levels of surface-displayed HaloTag (green in Fig. 2B). These fastest-growing strains, except sp0373, reached the highest levels of intracellular folded protein, particularly strain sp1643, for which the intracellular signal was similar to that of the strain expressing cytoplasmic HaloTag (Fig. 2B). On the other hand, the strains with the poorest growth, particularly sp0600, exhibited the highest levels of surface-displayed protein and low levels of intracellular protein (red in Fig. 2B). The difference in fluorescence corresponding to the amount of intracellularly folded protein (red) and surface-displayed protein (green) among Cyt, sp2940, sp1643, and sp0600 is clearly visible in the micrographs presented in Fig. 2C. Interestingly, while the HaloTag protein appears to be evenly distributed in the Cyt strain, it appears to be in foci located near the membrane for the translocating strains. These analyses thus suggest that strains with the most inhibited growth translocate and anchor more proteins, and vice versa.

In the experiments above, we used a final concentration of 25 ng/mL SppIP to achieve maximum expression levels, which is relevant when applying lactobacilli for vaccine delivery. To investigate the effects of overproduction of the LPxTG-anchored HaloTag protein on growth rate and surface display, the inducer concentration was reduced to 0.25 ng/mL SppIP (Fig. S4). Reduction of the inducer concentration generally led to improved growth, while considerable amounts of HaloTag were still produced. Expectedly, lowering the level of expressed protein, putting less stress on the secretion machinery, was beneficial for growth. Interestingly, for the “green” strains (high growth, low secretion efficiency), reduction of the inducer concentration led to markedly improved secretion efficiency (Fig. S4D). This shows that at lower protein production levels, the secretion machinery manages to translocate a larger fraction of the protein.

### The hydrophobicity of the signal peptide is a major determinant of growth performance and secretion efficiency

Knowing that the signal peptide is an important determinant of both growth and translocation efficiency, we conducted a Pearson's correlation analysis to investigate correlations between various characteristics of the signal peptides and the observations described above (Table S4). The analysis revealed several correlations, but only for the data set obtained when using the higher inducer concentration (Table 1). The analysis showed a positive correlation between the measured signal for surface-displayed protein (RFU AF488sd) and both the maximum hydrophobicity (*H*_max_) of the signal peptide and the length of the combined H and C domain of the signal peptide (*r* = 0.66 and 0.72, *r*^2^ = 43.6 and 51.8%, respectively, significant at *α* = 0.05). Interestingly, there was a negative correlation between the measured signal for intracellular folded protein (RFU TMRInt) and *H*_max_ of the signal peptide (*r* = −0.78, *r*^2^ = 60.8%, significant at *α* = 0.05). Taken together, the negative correlation between RFU TMRInt and *H*_max_ and the positive correlation between RFU AF488sd and *H*_max_ suggest that *H*_max_, i.e. signal peptide hydrophobicity, is important for keeping the preprotein in a state that is compatible with translocation. Most interestingly, the final significant correlation was a negative correlation (−0.76) between the signal for extracellular protein (RFU AF488sd) and growth, confirming trends described above showing that increased protein translocation hampers growth. Looking at the other correlations (Table 1), one would then assume to also find a negative correlation between *H*_max_ and growth, such a statistically significant correlation was indeed found.

**Table 1.** Pearson’s correlation analysis.

	AF488sd	TMRInt	OD_600_	*H*_max_	H + C-domains
AF488sd	1.00				
TMRInt	−0.47	1.00			
OD_600_	***−0***.***76***	0.44	1.00		
*H*_max_	***0***.***66***	***−0***.***78***	***−0**.**85***	1.00	
H + C-domains	***0***.***72***	−0.21	−0.52	0.33	1.00

The table shows the different parameters used and the resulting correlation coefficients (*r*) from the correlation analysis. Correlation coefficients highlighted in bold-italic are significant at *α* = 0.05.

**Fig. 3.**
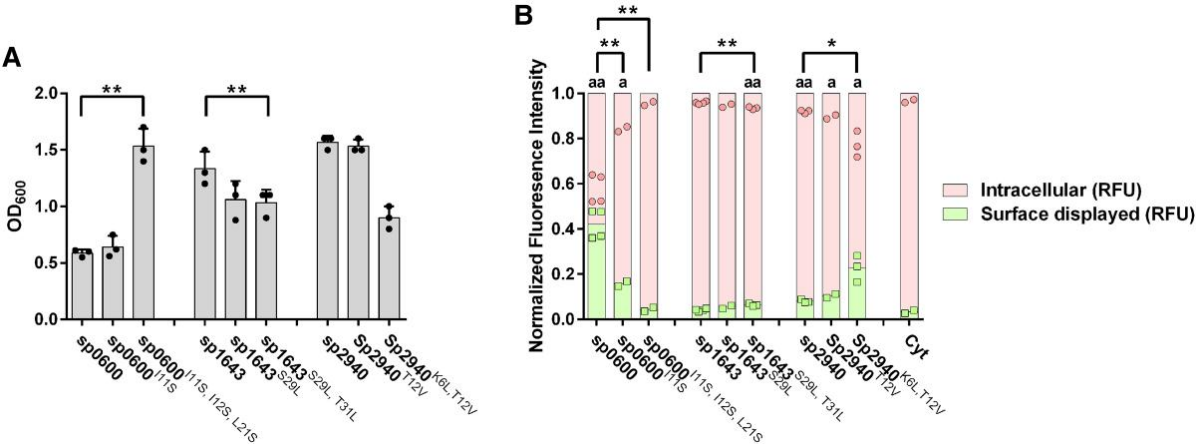
Characterization of recombinant *L. plantarum* strains expressing HaloTag anchored to the surface using the cwa3001 anchor, with either mutated or wild-type signal peptides. A) The OD_600_ 3 h after induction. B) Normalized RFUs of intracellular folded HaloTag protein (red, top) and folded protein anchored to the surface of the bacteria (green, bottom). The RFU values were normalized by dividing each of the fluorescent signals (intracellular and surface displayed) by the sum of both (intracellular + surface displayed). As a result, the sum of the two normalized values for each sample equals 1, allowing for comparison of the relative contributions of each signal type. The graph shows 2–4 biological replicates; all data points are shown. Statistical significance was analyzed using an independent, two-tailed t test. Significance between wild-type strains and the corresponding mutant strains is indicated by asterisks: *P < 0.05, **P < 0.01. Significance between the control strain “Cyt” and the translocating strains in indicated by letters: ^a^*P* < 0.05, ^aa^*P* < 0.01.

To further assess if and how the *H*_max_ of the signal peptides affects secretion, we constructed mutated versions of sp0600, sp1643, and sp2940 to increase (sp1643 and sp2940) or decrease (sp0600) the *H*_max_ value (Table 2). We assessed the effect of the signal peptide mutations by comparing growth and the levels of intra- and extracellular folded HaloTag for strains carrying the mutated or wild-type signal peptides (Fig. 3A and B). The MicrobeJ analysis showed that the mutants sp0600^I11S^ and sp0600^I11S,I12S,L21S^, with increasingly reduced *H*_max_, exhibited an increase in intracellular folded HaloTag compared with wild-type sp0600 (Fig. 3B); the RFU for intracellular protein increased significantly in sp0600^I11S^ and sp0600^I11S,I12S,L21S^, and the protein level on the surface of the single and triple mutant was significantly reduced (Fig. 3B). The triple mutant showed clearly improved growth (Fig. 3A), as one would expect based on the results described above, while the single mutant did not show such an effect. Along the same lines, for sp1643 (a signal peptide with low *H*_max_, displaying high intracellular protein levels), increasing the *H*_max_ from 1.99 to 2.46 or 2.96, in sp1643^S29L^ and sp1643^S29L,T31L^, respectively, resulted in decreased intracellular protein levels, increased levels of surface-displayed protein and a reduction in growth (Fig. 3A–C). The changes in the amount of intracellular and surface-displayed protein was significant between sp1643^S29L,T31L^ and the wild-type sp1643. A similar trend was observed for the double mutant of sp2940, where the *H*_max_ was increased to 3.16. This resulted in a significant reduction in the amount of intracellular protein and increase in surface-displayed protein, in addition to reduced growth (Fig. 3B). Notably, neither sp0600^I11S,I12S,L21S^, sp1643^S29L^, nor sp1643 were significantly different from Cyt. The correlations presented in Fig. 3 vary in strength (large effects for sp0600, lesser effects for the other two signal peptides), yet the overarching trend is clear and consistent: A higher *H*_max_ is associated with lower growth, lower levels of intracellular folded protein and higher levels of surface-displayed protein.

**Table 2.** H regions and *H*_max_ of wild-type and mutated signal peptides.

Strain	Sequence of H region	*H*_max_
sp0600	LLLLIIMSLGFCGMLLM	3.23
sp0600^I11S^	LLLL**S**IMSLGFCGMLLM	2.64
sp0600^I11S,I12S,L21S^	LLLL**SS**MSLGFCGM**S**LM	2.06
sp1643	WVAASITVFSVGIGLTFSQV	1.99
sp1643^S29L^	WVAA**L**ITVFSVGIGLTFSQV	2.46
sp1643^S29L,T31L^	WVAA**L**I**L**VFSVGIGLTFSQV	2.96
sp2940	ALKIVMGITMLTGGIMA	2.11
sp2940^T12V^	ALKIVMGI**V**MLTGGIMA	2.66
sp2940^K6L,T12V^	AL**L**IVMGI**V**MLTGGIMA	3.16

Mutated amino acids are highlighted in bold.

## Discussion

Our initial studies with antigen AgE6 coupled to signal peptide sp3050 and various LPxTG anchors showed only minor differences in bacterial growth (Fig. S1). The insignificant variation in growth suggests that the nature of the LPxTG anchor cannot explain the growth defects observed upon heterologous expression of LPxTG-anchored proteins in *L. plantarum.* This finding led us to investigate the impact of the signal peptide. A comparison of 11 signal peptides for secretion of LPxTG-anchored AgE6 showed a large variation in strain fitness (Fig. 1), indicating that fitness and, likely, secretion efficiency are primarily related to processes occurring prior to or during the translocation itself and not to sortase processing of the LPxTG motif. By exchanging the AgE6 with the HaloTag protein and keeping the LPxTG anchor, we established a method for simultaneous detection of intracellular and surface-displayed folded protein in bacteria. The method does not allow monitoring of intracellular unfolded protein, and it is important to note that proteins that are intimately associated with the secretion machinery but not yet translocated are unfolded.

Analyses of the 11 HaloTag-expressing strains with various signal peptides revealed an inverse correlation between bacterial growth and the amount of surface-displayed protein. Interestingly, the low levels of surface display in the faster-growing strains were accompanied by higher levels of intracellular folded protein. These correlations and observations suggest that in faster-growing strains, the interaction between the signal peptide and the secretion machinery is less efficient and that this is beneficial for the bacterium. An inefficient interaction may explain the observed increased intracellular HaloTag signal, which is likely due to premature intracellular folding and reduced translocation. At the same time, less efficient targeting of the secretion machinery would avoid overloading the secretion machinery with the heterologously produced protein. On the other hand, for signal peptides that strongly associate with the secretion machinery, one would then expect a lower intracellular HaloTag signal, due to reduced intracellular folding, whereas a higher burden on the secretion machinery could reduce fitness (38).

Our analysis revealed a positive correlation between the *H*_max_ of the signal peptide and the levels of intracellular and surface-displayed folded protein, indicating that high hydrophobicity is favorable for translocation to the surface. Subsequent mutational analyses with three signal peptides substantiated this correlation. The clearest results were obtained for sp0600: reducing *H*_max_ of sp0600 from 3.23 to 2.64 or 2.06 resulted in increasingly reduced surface display, higher levels of intracellular folded protein, and improved growth. The *H*_max_ of wild-type sp0600 is in the 90th percentile (>3.15) of *H*_max_ of all secreted proteins in *L. plantarum* WCFS1 (Fig. S5 (15)). Mutational increases in the *H*_max_ of sp1643 and sp2940 from 1.99–2.11 to 2.46–3.16 showed the expected tendencies, albeit less clearly than for sp0600. Interestingly, however, the largest effect was observed for the double mutant of sp2940 with an *H*_max_ of 3.16, i.e. the only one of four engineered variants with an *H*_max_ above 3.0. This mutant showed improved surface display, less intracellular protein, and decreased growth. Earlier studies have shown that only the ∼20% most hydrophobic signal peptides are targeted by SRP, whereas others are targeted by SecA (22). Our mutational data clearly show that high signal peptide hydrophobicity promotes translocation, similar to what has been observed in *E. coli* (39). One could speculate that high hydrophobicity leads to SRP targeting, which again leads to co-translational translocation, less accumulation of intracellular folded protein, and a larger extent of surface display. Of note, in *L. plantarum* WCFS1, the majority of secreted proteins carry signal peptides with an *H*_max_ in the range of 2.2–2.8 (Fig. S5) and are thus likely targeted by SecA.

As alluded to above, studies with *E. coli* have shown that the most hydrophobic signal peptides likely are recruited to the SRP-dependent co-translational secretion pathway rather than the SecA-dependent posttranslational secretion pathway (27, 39). Simultaneous translation and translocation, as it occurs in the co-translational SRP-dependent pathway, is thought to be important for keeping the prepeptides in the unfolded state (40). For proteins secreted via the posttranslational pathway in, e.g. *E. coli*, the SecB chaperone is important to inhibit prepeptide folding (41). Because *L. plantarum* WCFS1 does not encode SecB (11), proteins secreted via the SecA pathway may be even more at risk for premature folding, which, in the case of recombinant strains such as those studied here, may be good for cell fitness but not for obtaining efficient translocation and surface display.

Previous studies have rarely considered the growth performance of the producer strains and were largely focused on showing that the signal peptide is a major determinant of secretion efficiency (15, 16, 42). We observed that the growth varied among the 11 HaloTag strains, as it did among the AgE6 strains. The ranking of the strains by growth was similar but not identical for the two series of strains (Figs. 1A and 2A), showing that the nature of the secreted protein also plays a role. As outlined above, we propose that the impact of the signal peptide on growth performance of our recombinant *L. plantarum* strains relates to the extent to which the signal peptide “escapes” the secretion machinery. Such an escape would lead to the accumulation of intracellular folded protein and reduce the burden that an overexpressed heterologous protein may put on the secretion machinery. It is almost counterintuitive, but unequivocal based on the present results, that when expressing a secreted recombinant protein, more efficient secretion comes at the expense of fitness.

Interestingly, the four signal peptides giving the lowest translocation efficiency and the highest growth are derived from LPxTG proteins (sp2940, sp2796, sp0373, and sp1643; Fig. 2B). It would perhaps be reasonable to postulate that these signal peptides are optimal for translocation of recombinant LPxTG proteins as well, but our data show the opposite. Of course, promoter and gene expression levels in wild-type strains will be different from the levels in the recombinant strains used here, and the control experiment depicted in Fig. S4, shows that expression levels matter. The observed differences may also be attributed to the nature of the expressed protein, as it has been shown that the N-terminus of the mature protein has an impact on translocation (17), and since it is generally known from studies with overexpressed heterologous proteins that signal peptide efficiency is protein dependent in part (15, 16). The *H*_max_ of these four signal sequences was calculated to be the lowest among all studied signal peptides. Thus, these four LPxTG-protein-derived signal peptides likely do not lead to SRP recruitment.

Studies on the impact of protein production level and the levels of intracellular and surface-displayed folded HaloTag showed that tuning the protein production level affects premature folding, surface display, and fitness (Fig. S4). Reducing the amount of the inducer peptide, SppIP, led to increased growth for all strains and a general decrease in intracellularly folded protein, underpinning that translocation proceeds more efficiently and with a lower “secretion burden” on the cells. Two features stand out. Firstly, for the “green” strains (good growth, low surface display), a considerably higher fraction of folded HaloTag ended up on the cell surface. Secondly, upon reduction of the SppIP dose, the sp0600 strain, maintained high levels of surface-displayed HaloTag, while showing drastically improved growth, likely due to a lesser “seretion burden.”

## Conclusion

Our study uncovers key relationships between signal peptide characteristics, the surface display of LPxTG-anchored heterologous proteins, and growth performance in *L. plantarum*. By utilizing the HaloTag reporter protein, we demonstrate that achieving effective surface display of heterologous proteins incurs a significant fitness cost. Our findings suggest that highly hydrophobic signal peptides, which are likely to be recognized by the SRP, are particularly effective in preventing premature intracellular folding—a crucial requirement for successful translocation and surface anchoring. However, the associated secretion pathway appears prone to congestion, leading to reduced bacterial growth. These growth impairments highlight the need for a balanced approach when exploiting microbial fermentation for production and secretion of heterologous proteins, for instance, during development of bacterial delivery vehicle platforms, where optimizing protein expression levels could mitigate fitness deficits.

## Materials and methods

### Bacterial strains, plasmids, and growth conditions

*Escherichia coli* TOP10 was grown in brain–heart infusion (BHI; Oxoid Ltd, Basingstoke, United Kingdom) broth at 37 °C with shaking. *L. plantarum* WCFS1 was grown in MRS broth (Oxoid Ltd.), at 37 °C without shaking. Solid media were prepared by adding 1.5% (w/v) agar to the broth. When appropriate, cultures of *E. coli* or *L. plantarum* WCFS1 were supplemented with 200 or 10 µg/mL erythromycin, respectively.

### Construction of expression plasmids with different LPxTG anchors

Genomic DNA from *L. plantarum* WCFS1 was used as a template to amplify the LPxTG-anchor sequences listed in Table S1 with gene-specific primers (Table S2). All primers were designed to facilitate initial InFusion cloning and facilitate downstream cloning. The plasmid pLp_3050_Ag85B:ESAT6cwa2 was linearized by *Mlu*I/*Hind*III digestion, removing the cell wall anchor cwa2 (cwa2578). Subsequently, the PCR-amplified DNA fragments containing the new LPxTG anchors were inserted into the linearized vector following the InFusion protocol (Takara Bio, Kusatu, Japan), followed by transformation into chemically competent *E. coli* TOP10 cells (Invitrogen, Waltham, MA, United States), yielding pLp_3050-AgE6-cwa0373, pLp_3050-AgE6-cwa1643M, pLp_3050-Ag85B-E6-cwa1643L, pLp_3050-AgE6-cwa2940, pLp_3050-AgE6-cwa3001, and pLp_3050-AgE6-cwa1229 (Table S3). *Lactiplantibacillus plantarum* was made competent as described in Aukrust and Blom (43). All plasmids were sequenced before subsequent transformation into electrocompetent *L. plantarum* WCFS1.

### Construction of expression plasmids with different signal peptide sequences

The AgE6-cwa3001 fragment was excised from pLp_3050-AgE6-cwa3001 using *Sal*I/*Hind*III and inserted using Quick ligase (NEB) into *Sal*I/*Hind*III-digested plasmids encoding different signal peptide sequences (pLp0373_NucA, pLp0600_NucA, pLp2145_NucA, pLp3064_NucA, pLp3411_NucA, and pLp3676_NucA; Table S3). For the remaining four plasmids with new signal peptides, the fragment Ag85B-E6-cwa3001 was PCR-amplified using InFusion forward primer SP-1097-F/SP-1643-F/SP-2796-F/SP-2940-F and reverse primer SP-AgE6-R (Table S2), containing the *Sal*I/*Bsa*AI sites, and cloned into the *Sal*I/*Bsa*AI-digested plasmids pLp_1097-NucA, pLp_1643-NucA, pLp_2796-NucA, and pLp_2940-NucA (Table S3), using the InFusion cloning kit. A *Hind*III-restriction site downstream of the AgE6-cwa3001 fragments was inserted in the PCR to facilitate possible future cloning with these latter four plasmids. In total, 10 new plasmids with different signal peptides were constructed (Table S3), which were initially transformed to chemically competent *E. coli* TOP10 cells. All plasmids were sequenced before subsequent transformation into electrocompetent *L. plantarum* WCFS1.

### Construction of HaloTag-expressing plasmids

The *L. plantarum* codon-optimized sequence of HaloTag was ordered from ThermoFisher Scientific (Waltham, MA, United States), and amplified using primers HaloTag_*Sal*I_F/HaloTag_*Mlu*I_R. The PCR-amplified HaloTag and the 11 plasmids encoding different signal peptides and the cwa3001 cell wall anchor were digested with *Sal*I and *Mlu*I. The digested HaloTag amplicon was ligated into the linearized vector with Quick ligase, according to the manufacturer's protocol. The ligation mixtures were transformed into chemically competent *E. coli* TOP10 cells. All plasmids were sequenced before subsequent transformation to electrocompetent *L. plantarum* WCFS1.

### Mutation of signal peptides

Mutated versions of three signal peptides were made to assess the effect of hydrophobicity on secretion efficiency. To determine the *H*_max_ of the wild-type and mutated signal peptides, the ProtScale (44) web tool was used with a window size of nine residues.

Site-directed mutations in signal peptide sp0600 were made using PCR and primers designed to introduce a specific point mutation at residue 11 (I to S), or a triple mutation at positions 11, 12, and 21 (I11S, I12S, and L21S). The signal peptide was amplified with a forward primer with either the single or the triple mutation and Sp0600_SalI_R (Table S2). The mutated signal peptide was cloned into the *Nde*I/*Sal*I-digested vector (pLp_0600-HaloTag_cwa3001) using the InFusion kit (Takara), yielding pLp_sp0600^I11S^-HaloTag_cwa3001 and pLp_sp0600^I11S,I12S,L21S^-HaloTag_cwa3001.

Gene fragments of signal peptide sp1643 with a single mutation (S29L) or a double mutation (S29L, T31L), both flanked by *Nde*I and *Sal*I restriction sites, were ordered from ThermoFisher Scientific and cloned directly into the *Nde*I/*Sal*I-digested vector (pLp_1643-HaloTag_cwa3001) with the InFusion kit (Takara), yielding pLp_sp1643^S29L^-HaloTag_cwa3001 and pLp_sp1643^S29L,T31L^-HaloTag_cwa3001.

Site-directed mutations of the signal peptide sp2940 were made by amplification of the signal peptide with primer Sp2940_R and a forward primer containing either a single mutation at residue 12 (T to V) or a double mutation at residues 6 and 12 (K6L and T12V; Table S2). The mutated signal peptide was cloned into the *Nde*I/*Sal*I-digested vector (pLp_2940-HaloTag_cwa3001) with the InFusion kit (Takara), yielding sp2940^T12V^-HaloTag_cwa3001 and sp2940^K6L,T12V^-HaloTag_cwa3001.

The plasmids were first transformed into chemically competent *E. coli* TOP10 cells and sequenced before subsequent transformation into electrocompetent *L. plantarum* WCFS1.

### Growth and induction of gene expression

Overnight cultures of recombinant *L. plantarum* were diluted to an OD_600_ of ∼0.15 in prewarmed MRS. When the cultures reached an OD_600_ of ∼0.30, SppIP ((45); Caslo ApS, Lyngby, Denmark) was added to a final concentration of 25 ng/mL to induce maximum expression, unless otherwise stated. For all downstream analyses (sodium dodecyl sulfate–polyacrylamide gel electrophoresis [SDS–PAGE], western blot, flow cytometry, and microscopy), the bacteria were harvested 3 h after induction by centrifugation at 5,000 × *g* for 10 min at 4 °C. Before harvesting, OD_600_ values were determined.

### Protein extracts and SDS–PAGE

The harvested bacteria (from 10 mL of culture) were resuspended in 1 mL of PBS and transferred to FastPrep tubes containing 0.5 g acid washed glass beads (Sigma-Aldrich, Saint-Louis, MO, United States). The cells were lysed using a FastPrep FP120 Cell Disrupter (MP Biomedicals, Santa Ana, CA, United States), with three cycles of shaking at 6.5 m/s for 45 s, with 5-min pauses on ice between each run. After cell lysis, the samples were centrifuged at 16,000 × *g* for 1 min at 4 °C. The protein concentration of the cell-free, crude protein extract was measured using the Bradford Assay (Bio-Rad), with bovine serum albumine (BSA) as standard. In short, 5 µL sample, or PBS for the blank sample, and 795 µL dH_2_O were mixed with 200 µL protein assay reagent (Bio-Rad). The suspension was vortexed and incubated for 5 min at room temperature before measurement at 595 nm. Subsequently, the samples were prepared for SDS–PAGE analysis by mixing 20 µL lysate with 3 µL 10× NuPAGE Sample Reducing Agent and 7.5 µL 4× NuPAGE LDS Sample Buffer (both Invitrogen). After boiling the samples for 10 min, varying volumes of the samples, corresponding to 280 ng protein, were loaded onto the SDS–PAGE gel.

### Western blot

After the SDS–PAGE, the proteins were transferred to a nitrocellulose membrane using the iBlot Dry Blot System (Invitrogen). After blotting, visualization of the target protein was achieved using the SNAP i.d. 2.0 protein detection system (Sigma-Aldrich) following the manufacturer's protocol. The mouse monoclonal primary antibody anti-ESAT-6 (ab26246, Abcam, Cambridge, United Kingdom) was diluted 1:2,000 TBS (1.5 mM NaCl, 0.10 M Tris-HCl [pH 7.5]) containing 3% BSA, and the polyclonal horseradish peroxidase (HRP)-conjugated anti-mouse IgG secondary antibody (Sigma-Aldrich) was diluted 1:33,000 in TBS/3% BSA. The proteins were visualized using the SuperSignal West Pico PLUS Chemiluminescent substrate (ThermoFisher Scientific) and imaged with the Azure c400 system (Azure Biosystems, Dublin, CA, United States).

### Flow cytometry

Flow cytometric analysis was performed to assess the surface display of AgE6 in *L. plantarum* as described by Wiull et al (46). In short, after induction as described above, cultures (10 mL) were grown for three hours before the cells were harvested and washed once with 600 µL PBS. The bacterial cells were resuspended in 50 µL PBS (1.8 mM KH_2_PO_4_, 10 mM Na_2_HPO_4_, 137 mM NaCl, 2.7 mM KCl, pH 7.4) containing 2% (w/v) BSA and the 1:250 diluted anti-ESAT-6 antibody, followed by incubation for 30 min at room temperature. After washing the cells twice with 600 μL PBS/2% BSA, to remove the antibody, the cells were resuspended in 50 µL PBS containing 2% BSA and a 1:170 diluted fluorescein isothiocyanate (FITC)-conjugated anti-mouse IgG secondary antibody (Sigma-Aldrich), followed by incubation for 30 min at room temperature, protected from light. The bacteria were then washed three times with 600 μL PBS/2% BSA before subsequent analysis using a MACSQuant analyzer (Miltenyi Biotec GmbH, Bergisch Gladbach, Germany). The data were analyzed using FlowJo software (BD Bioscience, Franklin Lakes, NJ, United States).

### Microscopy and MicrobeJ single-cell image analysis

HaloTag expression was induced, as described above. Three hours after induction, a volume corresponding to $500\;\mu L/OD_{600}$ was harvested from each culture, after which the cells were pelleted (5,000 × *g*, 2 min, room temperature) and resuspended in a mixture of 50 µL PBS and 0.50 μL 1 mM AlexaFluor 488 ligand (Promega, Madison, WI, United States) per sample. The cell-impermeable AlexaFluor 488 stains surface-displayed HaloTag only. The cell suspensions were incubated at 37 °C for 15 min, before the excess ligand was removed by adding 500 μL PBS followed by centrifugation (5,000 × *g*, 2 min, room temperature). The cell pellet was then resuspended in a mixture of 50 μL PBS and 0.25 μL 0.1 mM TMRDirect to stain intracellular HaloTag. Multisample object glasses (Hendley-Essex, London, United Kingdom) were coated with 600 μL 1.2% agarose/PBS gel and loaded with 0.4 µL of each sample. The images were acquired with a Zeiss AxioObserver microscope equipped with an ORCA-Flash4.0 V2 digital CMOS camera (Hamamatsu Photonics, Hamamatsu City, Japan).

The microscopy images were analyzed using Fiji (47), and single-cell image analysis was performed with the MicrobeJ plugin extension (48). Multiple images (5–10 images, depending on the density of bacteria) of each strain were merged into three separate stacks, one for the phase contrast images, one for the green channel images (AlexaFluor 488) and one for the red channel images (TMRDirect). These stacks were loaded into MicrobeJ, and the area, length, and sinuosity were adjusted to fit the *L. plantarum* cells, enabling segmentation of single cells from the images. The detection of intensity maxima of the bacteria for both the green and the red channels separately were also included in the analysis. Based on this analysis, the median intensity (typically from >500 cells for each strain) from the red channel (corresponding to TMRDirect/intracellular protein) and the green channel (corresponding to AlexaFluor 488/surface-displayed protein) were determined.

### Statistical analysis

Independent two-tailed t tests were performed using Excel. The clustered dendrogram was generated using Jamovi software (The jamovi project (2024). *jamovi* (Version 2.5) [Computer Software]. Retrieved from https://www.jamovi.org).

## Supplementary Material

pgaf131_Supplementary_Data

## Data Availability

All data are included in the Supplementary material and source data files.
