# Supplementary material for: An inverse relationship between fitness and secretion efficiency in a gram-positive bacterium
Source: PNAS Nexus. 2025 Apr 28;4(5):pgaf131. doi: 10.1093/pnasnexus/pgaf131 (PMC12046399; doi:10.1093/pnasnexus/pgaf131)
Supplement: pgaf131_Supplementary_Data [file pgaf131_supplementary_data.docx]

**Supporting information for**

**An inverse relationship between fitness and secretion efficiency in a Gram-positive bacterium**

Kamilla Wiull^*^, Morten Kjos, Vincent G. H. Eijsink*, Geir Mathiesen

Faculty of Chemistry, Biotechnology and Food Science, NMBU - Norwegian University of Life Sciences, Ås, 1433, Norway

^*^To whom correspondence may be addressed. Email: vincent.eijsink@nmbu.no or kamilla.wiull@nmbu.no

**This PDF file includes:**

Figures S1-S5

Tables S1-S4

SI References


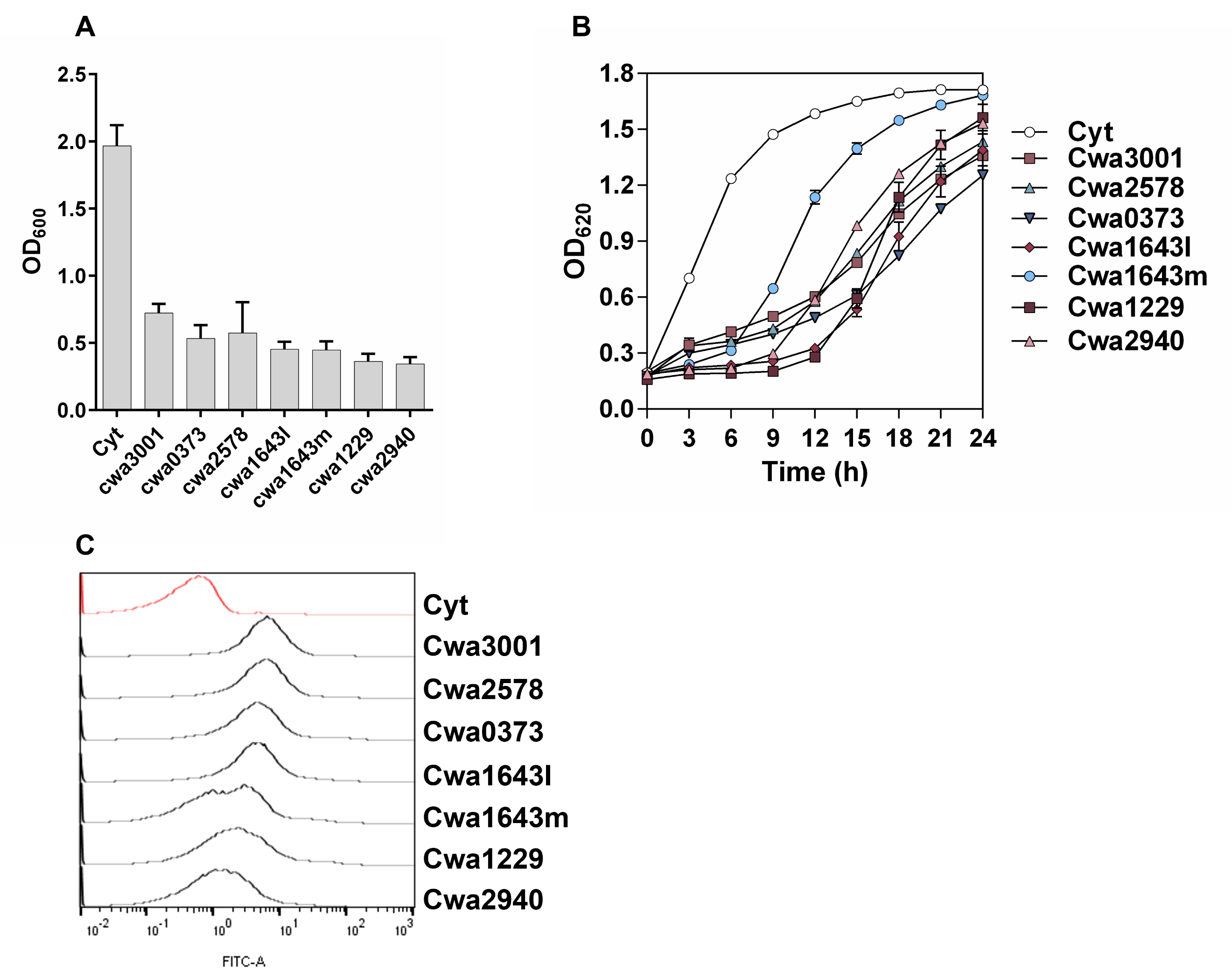


**Figure S1.** Characterization of recombinant *L. plantarum* strains expressing the AgE6 fusion antigen, targeted to the cytoplasm (“Cyt”) or targeted for cell-wall anchoring using seven different LPxTG-anchors. (A) OD_600_ of the strains at 3 hours post-induction of gene expression. The error bars show the standard deviation of three biological replicates. (B) Continuous measurement of the growth of recombinant *L. plantarum* strains producing and translocating cell wall anchored AgE6 antigen. The absorbance (620nm) was measured every 15 minutes for 24 hours using a ThermoScientific Multiscan FC. Error bars, representing the standard deviations of three biological replicates, are shown but often hidden by the symbols. Note that, eventually, all strains start growing well, because expression of the antigen, and with that secretion stress, is switched off. Also note that the strain labeled “cwa3001” is the same as the strain labeled “sp3050-Ag” in Fig. 1 of the main manuscript. (C) Flow cytometry analysis of surface display of AgE6 in cells harvested three hours post-induction, using an ESAT-6 specific primary antibody. Table S1 provides detailed information on the anchoring sequences.





**Figure S2.** Continuous measurement of the growth of recombinant *L. plantarum* strains producing and translocating cell wall anchored HaloTag. The absorbance (620nm) was measured every 10 minutes for 24 hours using a ThermoScientific Multiscan FC. Error bars, representing the standard deviations of three biological replicates, are shown at every three-hour interval and are mostly hidden by the symbols.


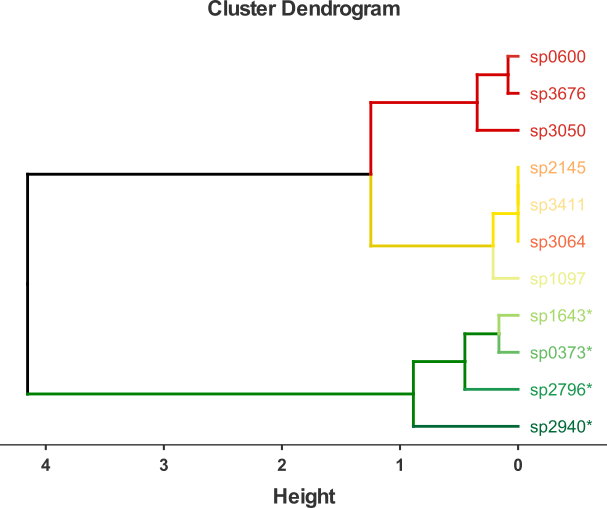


**Figure S3.** Grouping of the HaloTag secreting strains in a clustered dendrogram based on the OD_600_ three hours after induction. The clustered dendrogram was made in Jamovi, with the number of clusters set to three. The height (horizontal axis) shows that the intermediate and slow growing strains (yellow to red) have more similar OD_600_ values, and are distinctly different from the green strains.



**Figure S4.** Characterization of recombinant *L. plantarum* strains expressing HaloTag anchored to the surface using the cwa3001-anchor and various signal peptides. The cells were induced with 0.25 ng/ml SppIP (solid bars) or with 25 ng/ml SppIP (patterned bars) to obtain varying protein production levels. (A) OD_600_ of the strains, measured three hours after induction. (B) Single cell analysis of intracellular folded protein. (C) Single cell analysis of surface displayed folded protein. (D) The ratio of surface displayed and intracellular HaloTag signals (RFU AlexaFluor 488/(RFU TMRDirect+RFU AlexaFluor 488)). All analyses shown in panel A-D were performed three hours after induction. The error bars represent the standard deviations of three biological replicates. Day-to-day variation was high, explaining the large standard deviations. Each individual experiment showed the same trends, which are also visible in the Figure. The clearest of these is that for the faster-growing strains that show low secretion efficiency (Figs. 2 and 3 in the main manuscript; green color in Fig. 3 and above), reduction of the inducer dosage leads to improved secretion efficiency..


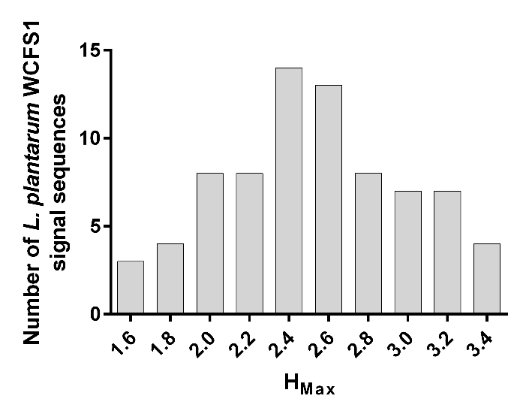


**Figure S5.** Histogram showing the distribution of maximum hydrophobicity (H_Max_) for all predicted signal peptides (n=76) in the *L. plantarum* WCFS1 genome (as determined by (1)). The bin width of each bar in the histogram is 0.2. The window size used to determine H_Max_ was nine.

**Table S1.** Overview of the LPxTG-anchors (cwa) and signal peptides used in this study. The table columns show the name, the locus tag from which the anchor or signal peptide originates, and the amino acid sequence. The LPxTG-motif is highlighted in bold; the amino acids of the mature cell wall anchor after predicted sortase processing are highlighted in italics and corresponds to the numbers in the parenthesis after each sequence. The amino acids downstream of the threonine in the LPxTG-motif are cleaved off by the sortase, and are not a part of the mature, anchored protein. The signal peptide sequences include the signal peptidase I cleavage site in bold (cleavage happens after the C-terminal A). For the mutated signal peptides, the mutated residues are marked in red.

| **Anchor name** | **Locus tag** | **Sequence** |
| --- | --- | --- |
| Cwa0373 | *lp_0373* | *GYAWAAENNDVTAAPASSATTSSESAASESNTNSSDSSRT*  *ASSAVDHSTSSASTSDASQSSHSTSSGESSHPESSSGSST*  *TSDSADADKQAAARSSQTQSNSVNGSSQAVSSSTVTSQSS*  *VPTKANTKQASSTPTTKANRATVAAATSSTAPRQSRATTA*  *SASVPSVTSASAVAASRDKQQSAFKKQHPILNQI****LPKT*N**S  AVATWLVWLGVGLLLLTVAITMVIKKRGRD (198) |
| Cwa1229 | *lp_1229* | *QFAAVDSPAIKGYTPDQSKISTQTVTGDSSDLEFTVVYKA*  *DSTSTKPVKPEQPTIPTTPTEPVKPGQLTTPAKPDQPMTS*  *DKSVQTITIKFVGQR****LPQT*N**ETDQQHMTLSGLLLLAMSGL  LGLLGMAKRQHKE (99) |
| Cwa1643M | *lp_1643* | *TQTVHYQSTTGTQLHDDTVRAMTFTRTKRVDQVTGDVTYS*  *NWSTNQADHTFERVAAFSIPGYHAVVTGTQAVMVTPASVD*  *DVQTIRYVTDRLSTGETPKTPVKTVTVNKSDKIKTTDTPD*  *KVATVKTPDKAQTVATTTAKQASVKRSVDLKQAQAVEQPA*  *QTRPANVKTVKLAKTTKSVKPTAAHQSATHKQAT****LPQT*N**D  DRQASVAAELLGLTAATLLVGVSAILKKRHN (198) |
| Cwa1643L | *lp_1643* | *LTGDYQTSSDYRTANTIADYSNQGYVLVRDSYPVSGAIFN*  *DDGVVHSYLVQLAHVTTATTETKTITQTVHYQSTTGTQLH*  *DDTVRAMTFTRTKRVDQVTGDVTYSNWSTNQADHTFERVA*  *AFSIPGYHAVVTGTQAVMVTPASVDDVQTIRYVTDRLSTG*  *ETPKTPVKTVTVNKSDKIKTTDTPDKVATVKTPDKAQTVA*  *TTTAKQASVKRSVDLKQAQAVEQPAQTRPANVKTVKLAKT*  *TKSVKPTAAHQSATHKQAT****LPQT*N**DDRQASVAAELLGLTA  ATLLVGVSAILKKRHN (263) |
| Cwa2578 | *lp_2578* | *VTEPGTTEPSKPGEPGTTEPSKPGEPGTTEPSKPGEPGTTEPSKPGEPGTTEPSKPGEPGTTEPSQPGEPGTTEPSKPDEPGTTEPSQPGKPGKPGEPGTTEPGNPGTTGPTAPQPERPAVPGPSQPAAPKPGQSGLGQPALPGLIKQPSTGVNGAGGTVGNGVTTGMNGFGTPTGSDQSTSAGYNHGT****LPQT*S**EKQSPIWVIFAGLLGLLIAAVGIGYRRRA (193) |
| Cwa2940 | *lp_2940* | *AGTQDQVVTAQRELSQAQAGLTTVRARTLATLTAAAEKPM*  *TEKPVGERPVVSHSTGTSTSTNQSAAPQATPAKPTLNQSS*  *SASVPTAQRVVTTQPRQATTVLRTTTSPAMAKPVTQQTVP*  *TTATKTAT****LPQT*G**EQTNRVLTVLGFVLLAATSLFGFSKQQ  RRHKTTD (132) |
| Cwa3001 | *lp_3001* | *ATADGTEVLVDLDNDNPLTSTAKPDENGSTTTKPDGNGTA*  *VKPDENGSTTTKPDGNGTAVKPDENGSTTTKPDGNGTAVK*  *PDENGSNTTKPGGNGTTVKPDKNGSSTTKPNGNGTAVKPD*  *KHETSTTGSGTVNTSGADKTSTNDNGTSMTAGTASSHAST*  *VTDRVTSGTVLPETSSSAATNHGSHSTGHHGSGW****LPQT*G**E  AVQRWLAVAGGVFLMLTGAIAVWWRKRRA (198) |
| **Signal peptide name** | **Locus tag** | **Sequence** |
| Sp0373 | *lp_0373* | MYTENTGKHHRNGLPVWLLPLLVVISFWGVSQNIMV**VDA** |
| Sp0600 | *lp_0600* | MMKHKNLLLLIIMSLGFCGMLLMFAPQISTLINPNASR**VEA** |
| Sp0600^I11S^ |  | MMKHKNLLLLSIMSLGFCGMLLMFAPQISTLINPNASR**VEA** |
| Sp0600^I11S, I12S, L21S^ |  | MMKHKNLLLLSSMSLGFCGMSLMFAPQISTLINPNASR**VEA** |
| Sp1097 | *lp_1097* | MKTKLIALIGVIAMVTGMAVFLNQRQQS**AKA** |
| Sp1643 | *lp_1643* | MRNRLNRLGLESKSHYKLYKSGRRWVAASITVFSVGIGLT  FSQVEQ**VKA** |
| Sp1643^S29L^ |  | MRNRLNRLGLESKSHYKLYKSGRRWVAALITVFSVGIGLT  FSQVEQ**VKA** |
| Sp1643 ^S29L,T31L^ |  | MRNRLNRLGLESKSHYKLYKSGRRWVAALILVFSVGIGLT  FSQVEQ**VKA** |
| Sp2145 | *lp_2145* | MKKINKLMILGMLVFGVTGATMINPEMTTA**AHA** |
| Sp2796 | *lp_2796* | MRLIVRSVRLFLKKWGITINYRESEVKCYKMYKSGKMWLL  ASASLLLLNTQLLT**AHA** |
| Sp2940 | *lp_2940* | MSKALKIVMGITMLTGGIMAQKMT**VHA** |
| Sp2940^T12V^ |  | MSKALKIVMGIVMLTGGIMAQKMT**VHA** |
| Sp2940^K6L,T12V^ |  | MSKALLIVMGIVMLTGGIMAQKMT**VHA** |
| Sp3050 | *lp_3050* | MKKFNFKTMLLLVLASCVFGVVVNVTTSLGPQTAIT**AQA** |
| Sp3064 | *lp_3064* | MRNRRVKWLLILVFGILGLLNLLPLE**GNA** |
| Sp3411 | *lp_3411* | MTKRMSFKFKWVALVATLIVGIGSWQVL**AHA** |
| Sp3676 | *lp_3676* | MRRLLTGTLVVGGLLLVVCLMAVN**GQA** |

**Table S2. Primers used in this study. Restriction sites appear in italics.**

| **Primer** | **Sequence (5’🡪3’)** | **Description** |
| --- | --- | --- |
| 1643cwa_F | CATGTTTGCA*ACGCGT*ACGCAAACCGTACATTATCA | Forward primer for amplification of *lp_1643* from the genome of *L. plantarum* WCFS1. Contains an *Mlu*I restriction site. |
| 1643cwa_R | CTGTAATTTG*AAGCTT*CTAATTATGACGTTTCTTCAAAATTGCA | Reverse primer for amplification of *lp_1643* from the genome of *L. plantarum* WCFS1. Contains a *Hind*III restriction site. |
| 0373cwa_F | CATGTTTGCA*ACGCGT*GGTTATGCGTGGGCAGCT | Forward primer for amplification of *lp_0373* from the genome of *L. plantarum* WCFS1. Contains an *Mlu*I restriction site. |
| 0373cwa_R | CTGTAATTTG*AAGCTT*TTAGTCACGCCCTCGTTTC | Reverse primer for amplification of *lp_0373* from the genome of *L. plantarum* WCFS1. Contains a *Hind*III restriction site. |
| 1229cwa_F | CATGTTTGCA*ACGCGT*CAATTTGCGGCCGTGGATA | Forward primer for amplification of *lp_1229* from the genome of *L. plantarum* WCFS1. Contains an *Mlu*I restriction site. |
| 1229cwa_R | CTGTAATTTG*AAGCTT*CTACTCTTTGTGCTGTCGC | Reverse primer for amplification of *lp_1229* from the genome of *L. plantarum* WCFS1. Contains a *Hind*III restriction site. |
| 1643cwa_short_F | GCATGTTTGCA*ACGCGT*AAGCCAACCGCGGCTC | Forward primer for amplification of *lp_1643* from the genome of *L. plantarum* WCFS1. The amplicon obtained with this forward primer results in a shorter anchor sequence compared amplification with 1643cwa_F. Contains an *Mlu*I restriction site. |
| 1643cwa_long_F | CATGTTTGCA*ACGCGT*TTGACTGGTGACTATCAGAC | Forward primer for amplification of *lp_1643* from the genome of *L. plantarum* WCFS1. The amplicon obtained with this forward primer results in a longer anchor sequence compared amplification with 1643cwa_F. Contains an *Mlu*I restriction site. |
| 3001cwa_F | CATGTTTGCA*ACGCGT*GCCACTGCCG ACGGTACA | Forward primer for amplification of *lp_3001* from the genome of *L. plantarum* WCFS1. Contains an *Mlu*I restriction site. |
| 3001cwa_R | CTGTAATTTGA*AGCTT*CTAGGCGCGTCGCTTCC | Reverse primer for amplification of *lp_3001* from the genome of *L. plantarum* WCFS1. Contains a *Hind*III restriction site. |
| 2940cwa_F | CATGTTTGCA*ACGCGT*GCGGGTACTCAAGATCAGGT | Forward primer for amplification of *lp_2940* from the genome of *L. plantarum* WCFS1. Contains an *Mlu*I restriction site |
| 2940cwa_R | CTGTAATTTG*AAGCTT*TTAATCAGTTGTTTTATGGCGC | Reverse primer for amplification of *lp_2940* from the genome of *L. plantarum* WCFS1. Contains a *Hind*III restriction site. |
| SP-1097-F | AGCGACGACC*GTCGAC*TTTAGTCGTCCAG | Forward primer for amplification of AgE6_cwa3001 from pLp_3050_Ag85B:ESAT6cwa2, with a 15 bp overhang complementary to pLp1097_NucA. Contains a *Sal*I restriction site |
| SP-1643-F | AGCCGCAACG*GTCGAC*TTTAGTCGTCCAG | Forward primer for amplification of AgE6_cwa3001 from pLp_3050_Ag85B:ESAT6cwa2, with a 15 bp overhang complementary to pLp1643_NucA. Contains a *Sal*I restriction site |
| SP-2796-F | TGCGGATGAA*GTCGAC*TTTAGTC GTCCAG | Forward primer for amplification of AgE6_cwa3001 from pLp_3050_Ag85B:ESAT6cwa2, with a 15 bp overhang complementary to pLp2796_NucA. Contains a *Sal*I restriction site |
| SP-2940-F | TGCAGCCGAA*GTCGAC*TTTAGT CGTCCAG | Forward primer for amplification of AgE6_cwa3001 from pLp_3050_Ag85B:ESAT6cwa2, with a 15 bp overhang complementary to pLp2940_NucA. Contains a *Sal*I restriction site |
| SP-AgE6-R(*Bsa*AI) | CAATCAAAGCAA*CACGTG*CTGTAATTTG*AAGCTT*CTAG | Reverse primer for amplification of AgE6_cwa3001 from pLp_3050_Ag85B:ESAT6cwa2, with a 15 bp overhang complementary to a *Bsa*AI-digested pSIP-plasmid. Additionally, for downstream use of the plasmid, a HindIII site is inserted. |
| SP-AgE6-R(*Hind*III) | CTGTAATTTG*AAGCTT*CTAGGCGCGTCGCTTCCGC | Reverse primer for amplification of AgE6_cwa3001 from pLp_3050_Ag85B:ESAT6cwa2, with a 15 bp overhang complementary to a *Hind*III-digested pSIP-plasmid. |
| HaloTag_*Sal*I_F | AGGCCTCCAAG*GTCGAC*GCTGAAATTGGTACGGGTT | Forward primer for amplification of the HaloTag. Contains a *Sal*I restriction site. |
| HaloTag_*Mlu*I_R | TCGGCAGTGGC*ACGCGT*ACCACTGATTTCTAACGTACT | Reverse primer for amplification of the HaloTag. Contains an *Mlu*I restriction site. |
| HaloTag_NdeI_F | AGGAGTATGATT*CATATG*GCTGAAATTGGTACGGGTT | Forward primer for amplification of the HaloTag, for construction of cytoplasmic HaloTag-expression. Contains a *Nde*I restriction site. |
| HaloTag_HindIII_R | GCTGTAATTTG*AAGCTT*TTAACCACTGATTTCTAACGTACT | Reverse primer for amplification of the HaloTag, for construction of cytoplasmic HaloTag-expression. Contains a *Hind*III restriction site. |
| S-mutant_sp0600_F | GGAGTATGATT*CATATG*ATGAAACATAAAAATTTATTATTATTAAGTATAATGAGCTTGGGTTTCTGC | Forward primer for inserting the I11S mutation in sp0600. Contains a *Nde*I restriction site. |
| SSS-mutant_sp0600_F | GGAGTATGATT*CATATG*ATGAAACATAAAAATTTATTATTATTAAGTAGTATGAGCTTGGGTTTCTGCGGGATGAGTTTGATGTTTGCGCCACAAATCA | Forward primer for inserting the I11S, I12S and L21S mutations in sp0600. Contains a *Nde*I restriction site. |
| Sp0600_SalI_R | CAATTTCAGCGTCGACCTTATCCGCTTCAACTCG | Reverse primer for amplification of the sp0600-mutants. Contains a *Sal*I restriction site. |
| Vsp2940_F | GGAGTATGATTCATATGTCAAAAGCGCTTAAGATAGTGATGGGAATCGTC ATGTTAACAGGGGGCATCAT | Forward primer for inserting the S29L mutation in sp2940. Contains a *Nde*I restriction site. |
| LVsp2940_F | GGAGTATGATTCATATGTCAAAAGCGCTTCTGATAGTGATGGGAATCGTC ATGTTAACAGGGGGCATCAT | Forward primer for inserting the S29L and T31L mutations in sp2940. Contains a *Nde*I restriction site. |
| Sp2940_R | CAATTTCAGCGTCGACTTCGGCTGCATGTACCGT | Reverse primer for amplification of the sp2940-mutants. Contains a *Sal*I restriction site. |

**Table S3. Strains and plasmids used in this study.**

| **Plasmid** | **Nickname** | **Description** | **Reference** |
| --- | --- | --- | --- |
| pEV |  | Ery^r^; 256_rep_; pSIP401 derivative; control plasmid (“empty vector”) | (2) |
| pLp_cyt:AgE6-DC | Cyt-Ag | Ery^r^; 256_rep_; pSIP401 derivative; containing the inducible P*_sppA_* fused to a gene construct encoding Ag85B-ESAT-6 followed by a dendritic cell binding sequence (DC). | (3) |
| pLp_3050_Ag85B:ESAT6cwa2 | Cwa2578 | Ery^r^, 256^rep^; pSIP401 derivative; containing the inducible P*_sppA_* fused to a gene construct encoding the N-terminal signal peptide from the gene *lp_3050* followed by the sequence encoding Ag85B-ESAT-6 and a C-terminal LPxTG anchor derived from the gene *lp_2578.* This anchor is called cwa2578 in this study. | (4) |
| pLp_3050-AgE6-cwa0373 | Cwa0373 | Ery^r^, 256^rep^; pSIP401 derivative; containing the inducible P*_sppA_* fused to a gene construct encoding the N-terminal signal peptide from the gene *lp_3050* followed by the sequence encoding Ag85B-ESAT-6 and a C-terminal LPxTG anchor derived from the gene *lp_0373.* | This study |
| pLp_3050-AgE6-cwa1643 | Cwa1643 | Ery^r^, 256^rep^; pSIP401 derivative; containing the inducible P*_sppA_* fused to a gene construct encoding the N-terminal signal peptide from the gene *lp_3050* followed by the sequence encoding Ag85B-ESAT-6 and a C-terminal LPxTG anchor derived from the gene *lp_1643.* | This study |
| pLp_3050-AgE6-cwa1643M | Cwa1643m | Ery^r^, 256^rep^; pSIP401 derivatives; containing the inducible P*_sppA_* fused to a gene construct encoding the N-terminal signal peptide from the gene *lp_3050* followed by the sequence encoding Ag85B-ESAT-6. The C-terminal LPxTG-anchor in both plasmids is derived from the gene *lp_1643,* but differs in linker length (194 residues for medium (M) and 259 residues for long (L)) between the anchor and the antigen. | This study |
| pLp_3050-AgE6-cwa1643L | Cwa1643l |  |  |
| pLp_3050-AgE6-cwa2940 | Cwa2940 | Ery^r^, 256^rep^; pSIP401 derivative; containing the inducible P*_sppA_* fused to a gene construct encoding the N-terminal signal peptide from the gene *lp_3050* followed by the sequence encoding Ag85B-ESAT-6 and a C-terminal LPxTG anchor derived from the gene *lp_2940.* | This study |
| pLp_3050-AgE6-cwa3001 | Cwa3001/sp3050-Ag (Figure S1/Figure 1) | Ery^r^, 256^rep^; pSIP401 derivative; containing the inducible P*_sppA_* fused to a gene construct encoding the N-terminal signal peptide from the gene *lp_3050* followed by the sequence encoding Ag85B-ESAT-6 and a C-terminal LPxTG anchor derived from the gene *lp_3001.* | This study |
| pLp_3050-AgE6-cwa1229 | Cwa1229 | Ery^r^, 256^rep^; pSIP401 derivative; containing the inducible P*_sppA_* fused to a gene construct encoding the N-terminal signal peptide from the gene *lp_3050* followed by the sequence encoding Ag85B-ESAT-6 and a C-terminal LPxTG anchor derived from the gene *lp_1229*. | This study |
| pLp0373_NucA |  | Ery^r^, 256^rep^; pSIP401 derivatives; containing the inducible P*_sppA_* fused to a gene construct encoding *nucA* and a signal peptide derived from a *L. plantarum* protein, indicated by the gene number. | (1) |
| pLp0600_NucA |  |  |  |
| pLp1097_NucA |  |  |  |
| pLp1643_NucA |  |  |  |
| pLp2145_NucA |  |  |  |
| pLp2796_NucA |  |  |  |
| pLp2940_NucA |  |  |  |
| pLp3064_NucA |  |  |  |
| pLp3411_NucA |  |  |  |
| pLp3676_NucA |  |  |  |
| Plasmids used to generate results depicted in Figure 1 (encoding the Ag85B:ESAT6 antigen) | | | |
| pLp_0373-AgE6-cwa3001 | sp0373-Ag | Ery^r^, 256^rep^; pSIP401 derivatives; containing the inducible P*_sppA_* fused to a gene construct encoding a signal peptide derived from a *L. plantarum* protein, indicated by the gene number, followed by the sequence encoding Ag85B-ESAT-6 and a C-terminal LPxTG anchor derived from the gene *lp_3001* | This study |
| pLp_0600-AgE6-cwa3001 | sp0600-Ag |  |  |
| pLp_1097-AgE6-cwa3001 | sp1097-Ag |  |  |
| pLp_1643-AgE6-cwa3001 | sp1643-Ag |  |  |
| pLp_2145-AgE6-cwa3001 | sp2145-Ag |  |  |
| pLp_2796-AgE6-cwa3001 | sp2796-Ag |  |  |
| pLp_2940-AgE6-cwa3001 | sp2940-Ag |  |  |
| pLp_3064-AgE6-cwa3001 | sp3064-Ag |  |  |
| pLp_3411-AgE6-cwa3001 | sp3411-Ag |  |  |
| pLp_3676-AgE6-cwa3001 | sp3676-Ag |  |  |
| Plasmids used to generate the results depicted in Figures 2 & 3 (encoding the HaloTag) | | | |
| pLp_0373-HaloTag_cwa3001 | sp0373 | Ery^r^, 256^rep^; pSIP401 derivatives; containing the inducible P*_sppA_* fused to a gene construct encoding a signal peptide derived from a *L. plantarum* protein, indicated by the gene number, followed by the *L. plantarum optimized* sequence encoding a HaloTag and a C-terminal LPxTG anchor derived from the gene *lp_3001* | This study |
| pLp_0600-HaloTag_cwa3001 | sp0600 |  |  |
| pLp_1097-HaloTag_cwa3001 | sp1097 |  |  |
| pLp_1643-HaloTag_cwa3001 | sp1643 |  |  |
| pLp_2145-HaloTag_cwa3001 | sp2145 |  |  |
| pLp_2796-HaloTag_cwa3001 | sp2796 |  |  |
| pLp_2940-HaloTag_cwa3001 | sp2940 |  |  |
| pLp_3050-HaloTag_cwa3001 | sp3050 |  |  |
| pLp_3064-HaloTag_cwa3001 | sp3064 |  |  |
| pLp_3411-HaloTag_cwa3001 | sp3411 |  |  |
| pLp_3676-HaloTag_cwa3001 | sp3676 |  |  |
| pLp_Cyt-HaloTag | Cyt | Ery^r^, 256^rep^; pSIP401 derivative; containing the inducible P*_sppA_* fused to a gene construct encoding cytoplasmatic expression of the HaloTag. | This study |
| pLp_sp0600^I11S^-HaloTag_cwa3001 | sp0600^I11S^ | Ery^r^, 256^rep^; sp0600-HaloTag_cwa3001 derivative; containing the I11S mutation in the signal peptide. | This study |
| pLp_sp0600^I11S, I12S, L21S^-HaloTag_cwa3001 | sp0600^I11S, I12S, L21S^ | Ery^r^, 256^rep^; sp0600-HaloTag_cwa3001 derivative; containing the I11S, I12S and L21S mutations in the signal peptide | This study |
| pLp_sp1643^S29L^-HaloTag_cwa3001 | sp1643^S29L^ | Ery^r^, 256^rep^; sp1643-HaloTag_cwa3001 derivative; containing the S29L mutation in the signal peptide. | This study |
| pLp_sp1643^S29L, T31L^-HaloTag_cwa3001 | sp1643^S29L, T31L^ | Ery^r^, 256^rep^; sp1643-HaloTag_cwa3001 derivative; containing the S29L and T31L mutations in the signal peptide. | This study |
| pLp_sp2940^T12V^-HaloTag_cwa3001 | sp2940^T12V^ | Ery^r^, 256^rep^; sp2940-HaloTag_cwa3001 derivative; containing the T12V mutation in the signal peptide. | This study |
| pLp_sp2940^K6L,T12V^-HaloTag_cwa3001 | sp2940^K6L,T12V^ | Ery^r^, 256^rep^; sp2940-HaloTag_cwa3001 derivative; containing the K6L and T12V mutations in the signal peptide. | This study |
| **Strain** |  | **Description** | **Reference** |
| *Escherichia coli* TOP10 |  | Subcloning strain | Invitrogen |
| *Lactiplantibacillus plantarum* WCFS1 |  | Host strain | (5) |

**Table S4.** Values for each attribute used in the Pearson’s correlation analysis (excluding the signal peptide column). All attributes meet the assumption of normality for at least one of the compared attributes, based on verification using the Shapiro-Wilk analysis. The values in the columns RFU AF488sd, RFU TMRInt and OD_600_ represent means from three independent measurements. The values are derived from the experiments shown in Figure 2.

| **Signal peptide** | **RFU AF488sd** | **RFU TMRInt** | **OD_600_** | **H_Max_** | **Length of the H- plus the C-domain** | |
| --- | --- | --- | --- | --- | --- | --- |
| Sp2940 | 627 | 6923 | 1.7 | 2.11 | | 21 |
| Sp2796 | 575 | 5888 | 1.6 | 2.33 | | 21 |
| Sp0373 | 776 | 2317 | 1.4 | 2.86 | | 28 |
| Sp1643 | 650 | 15391 | 1.4 | 1.99 | | 25 |
| Sp1097 | 1089 | 4141 | 1.0 | 3.17 | | 27 |
| Sp3411 | 1033 | 4654 | 0.9 | 3.07 | | 21 |
| Sp2145 | 878 | 7146 | 0.9 | 2.92 | | 27 |
| Sp3064 | 972 | 2873 | 0.9 | 3.42 | | 22 |
| Sp3050 | 1007 | 4472 | 0.8 | 3.01 | | 32 |
| Sp3676 | 1035 | 2698 | 0.6 | 3.36 | | 24 |
| Sp0600 | 1887 | 2798 | 0.6 | 3.23 | | 36 |

**References**

1. G. Mathiesen *et al.*, Genome-wide analysis of signal peptide functionality in *Lactobacillus plantarum* WCFS1. *BMC Genomics.* **10**, 425 (2009).

2. L. Fredriksen *et al.*, Surface display of N-terminally anchored invasin by *Lactobacillus plantarum* activates NF-κB in monocytes. *Appl. Environ. Microbiol.* **78**, 5864 (2012).

3. K. Wiull *et al.*, Comparison of the immunogenic properties of *Lactiplantibacillus plantarum* carrying the mycobacterial Ag85B-ESAT-6 antigen at various cellular localizations. *Front. Microbiol.* **13**, 2114 (2022).

4. K. Kuczkowska *et al.*, Immunogenic properties of *Lactobacillus plantarum* producing surface-displayed *Mycobacterium tuberculosis* antigens. *Appl. Environ. Microbiol.* **83**, 02782 (2017).

5. M. Kleerebezem *et al.*, Complete genome sequence of *Lactobacillus plantarum* WCFS1. *Proc. Natl. Acad. Sci. U.S.A.* **100**, 1990 (2003).
